# Peripheral Neuropathy in Beta-Thalassemia: Corneal Confocal Microscopy-Based Study

**DOI:** 10.7759/cureus.32122

**Published:** 2022-12-01

**Authors:** Saad A Khan, Syed Ali H Naqvi, Amber Saeed, Wajid A Khan, Muhammad A Moqeet, Warda Ali, Faheem U Khan

**Affiliations:** 1 Cornea and Refractive Surgery, Al-Shifa Trust Eye Hospital, Rawalpindi, PAK

**Keywords:** nerve density, corneal nerves, confocal microscopy, peripheral neuropathy, beta-thalassemia

## Abstract

Background

Peripheral neuropathy is a controversial but serious complication of beta-thalassemia (β-Th). Although few studies have reported no relationship between neuropathy and thalassemia, many have linked it with increasing age, iron overload, and iron chelator toxicity. This study aims to investigate the presence of neuropathy in β-Th using corneal nerve fibers.

Methodology

A cross-sectional study was conducted using corneal confocal microscopy on individuals with intermediate and major β-Th who were compared to healthy individuals. The main outcome variables were corneal main nerve and branch nerve densities which were calculated using Image J software. The comparison between groups was done using the independent-samples F-test and Bonferroni post-hoc analysis.

Results

There was reduced corneal main nerve and branch nerve density in β-Th intermediate and major patients compared to the control group, and the results were statistically significant (p-value <0.05). However, a significant correlation was not observed between serum ferritin levels and corneal nerve parameters.

Conclusions

The reduction in corneal nerve parameters in β-Th patients compared to healthy controls can be an indication of peripheral neuropathy in β-Th. Further work is needed to confirm these findings.

## Introduction

Thalassemia is a group of hematological genetic disorders characterized by structural abnormality in hemoglobin, which leads to the impairment of its function [1]. Thalassemia is usually classified as the alpha or beta variant based on the chain of hemoglobin (alpha or beta chain) undergoing structural defects, with the alpha type associated with relatively early mortality. Beta-thalassemia (β-Th) is the most common inherited hemoglobin disorder in Pakistan, with as many as 5,000-9,000 cases of β-Th identified in the country every year [2]. Further, evidence indicates that around 10,000 individuals with β-Th in Pakistan are dependent on blood transfusions [3].

In addition to the basic blood-based pathology, β-Th patients suffer from a range of other complications. Regular red blood cell transfusions can lead to iron overload and deposition in different organs of the body. Moreover, the overstimulation of bone marrow and defective erythropoiesis lead to multiple complications [4]. A wide range of skeletal abnormalities may also develop, such as reduction in bone density, malocclusion of teeth, and increased susceptibility to fractures [5,6]. While there are very limited reports in childhood, neurological abnormalities, such as anomalies in visual, auditory, and somatosensory evoked potentials along with nerve conduction velocities, have been reported. Although the complete etiology of these complications is not understood, multiple factors such as chronic hypoxia, iron overload, deferoxamine neurotoxicity, and born marrow expansion have been associated with neurological complications [7].

Peripheral neuropathy associated with β-Th has been reported over the years, with up to 78% suffering from mild sensory neuropathy according to a previous study [8]. However, there have been contrasting results from studies in recent years concerning its association with β-Th. While a few studies have reported a relatively higher incidence of neuropathy in β-Th [9], others reported no association between them [10,11]. Nevertheless, these studies were done on children, and there is evidence that neuropathy does not develop until adolescence. All of these studies focused on the assessment of major nerves through traditional methods and did not focus on small nerve fibers which may be involved during the earlier stages of pathology as seen for other peripheral neuropathies associated with other diseases [12,13]. Although skin biopsy is considered the gold standard for detecting small fiber neuropathy [14], its invasive nature makes it inadequate in children and for screening purposes.

Corneal confocal microscopy (CCM) has been recently reported as an effective tool for early diagnosis and monitoring of peripheral neuropathy because of its ability to identify changes in small nerves [15,16]. The cornea is the most sensitive tissue of the body as it has the highest density of innervation [17]. Therefore, because of its non-invasive nature and relatively painless examination, CCM is an ideal tool for studying changes in corneal nerves and the development of markers for neurodegenerative diseases. It is being effectively used for clinical and research purposes in various systemic and neurological conditions, including diabetic neuropathy [18], Parkinson’s disease [19], and Alzheimer’s disease [20], among others. However, to our knowledge, it has not been used to study corneal nerve changes as markers of peripheral neuropathy in β-Th.

The goal of this study is to determine differences, if any, in corneal nerve parameters using CCM between thalassemia patients and healthy controls, as well as their relationship with tear film breakup time and serum ferritin levels.

## Materials and methods

A cross-sectional study was conducted on individuals having major and intermediate clinical variants of β-Th at the Department of Cornea and Refractive Surgery, Al-Shifa Trust Eye Hospital (ASTEH), Rawalpindi, Pakistan. The study was conducted after obtaining ethical approval from the Ethical Review Committee of the hospital (reference number: ERC-29/AST-22). Written informed consent was taken from all respondents or their guardians before inclusion in the study. Respondents had complete freedom to leave the study at any point in time. All procedures and methods of data collection were conducted following the Declaration of Helsinki. The study population was β-Th patients registered in the Thalassemia Center, Rawalpindi, Pakistan. A minimum age limit of 10 was selected because peripheral neuropathy has been observed to occur after adolescence according to some evidence in the literature. The data were obtained through consecutive non-probability sampling. Individuals with a negative family history of neuropathy, absence of any anatomical and metabolic cause for neuropathy, and negative history of any acute febrile illness within the previous three weeks were included in the study. Further, individuals with a history of autoimmune diseases, immunosuppression, neoplastic conditions, any ocular condition affecting corneal nerve integrity, and those on antioxidant supplementation therapy were excluded.

Data collection procedure

Data collection was done among all individuals visiting ASTEH for regular eye examinations following recommendations from their physicians in the Thalassemia Center. A thorough ophthalmic examination including clinical refraction was completed before data collection. Medical history was reviewed for every participant to ensure that inclusion and exclusion criteria were not ignored.

The protocol for image acquisition was based on a recent study [21]. The imaging was done through the Cornea module of Heidelberg Retinal Tomographer-3 (Heidelberg Engineering, Inc. Heidelberg, Germany) Images were obtained at two intervals separated by one week. A drop of proparacaine hydrochloride (0.5%) was instilled to anesthetize the eyes before examination along with a coupling agent which was instilled in the eye and the applanation cap. Patients were advised to fixate on the outer fixation light and a charge-coupled device-based camera was used to observe the eye’s fixation throughout the examination. The images were taken in the section mode of CCM with the 400-µm field lens. Six (three left eye (LE), three right eye (RE)) high-clarity images at 1-mm intervals from the central cornea of each subject were obtained in the sub-basal nerve plexus. Data were also collected from age-matched healthy individuals for comparison. Complete data collection was done by a single examiner.

Outcome parameters

The outcome of the study was corneal nerve fiber morphology which was assessed through nerve fiber density and branch fiber density in every picture selected for the analysis. These parameters have been established to be indicative of peripheral neuropathy [22]. The nerve fiber density was measured as the total length of nerve fibers/total area under observation, and the branch fiber density was measured as the total number of branches/total area under observation [21,22].

Image and data analysis

Image analysis was done using Image J software (National Institute of Health and the Laboratory for Optical and Computational Instruments, USA) using its Neuron J plugin. Data were analyzed by two independent observers masked from each other. In cases of conflict, a third observer was added and the final reading was selected after a discussion between the three examiners. The images were captured and saved as 384 × 484 pixel BMP images, which were cropped to 384 × 384 pixels, outlining only the 400 µm × 400 µm area using the Image J software. After calibrating pixel length with actual length, images were imported in Neuron-J software and magnified to 150%. Thereafter, semi-automatic tracing was done with the said plugin [23].

Complete analysis of data was conducted in SPSS version 21 (IBM Corp., Armonk, NY, USA). Rigorous data cleaning was done before the final analysis. In the descriptive analysis, frequencies were described for all categorical variables. Continuous variables were presented as mean along with the standard deviation (SD) where applicable.

In inferential analysis, all statistical tests utilized a confidence interval of 95%, and a p-value of <0.05 was considered significant. The independent-sample analysis of variance (ANOVA) test was conducted to determine the statistical significance of the difference in the nerve parameters among the individuals with thalassemia major, intermediate, and healthy subjects. Intergroup comparisons were done through post-hoc Bonferroni correction.

## Results

A total of 20 individuals with β-Th major, 14 with β-Th intermediate, and 20 age-matched controls were included in the study.

In total, 64 eyes of thalassemic patients were included in the analysis, of which 36 (34.6%) were associated with thalassemia major and the remaining 28 (26.9%) with thalassemia intermediate. A total of 40 (38.5%) eyes from healthy controls were also included in the study A majority of the sample comprised females (66.67%, n = 36) and the rest were males (33.33%, n = 18). The average age for the intermediate, major, and control groups was 20.43 ± 8.73 years, 17.65 ± 3.82 years, and 19.05 ± 5.43 years, respectively, and there was no statistically significant difference between the groups (p = 0.40). The average duration of the disease was 16.57 ± 7.98 and 16.45 ± 3.79 years for the intermediate and major groups, respectively. The demographic details and medical history are provided in Table 1.

**Table 1:** Background variables.

Variables	Intermediate group	Major group	Control group	P-value
	Mean ± standard deviation (range)			
Age (years)	20.43 ± 8.73 (10–36)	17.65 ± 3.82 (13–27)	19.15 ± 5.23 (10–27)	0.40
Serum ferritin levels (ng/mL)	2,266.71 ± 1,651.42 (350–5,000)	2,666.35 ± 1,703.33 (160–5,000)	-	0.50
Duration of disease (years)	16.57 ± 7.98 (1-28)	16.45 ± 3.79 (12–26)	-	0.95

All patients were on either oral, intravenous, or combined iron chelation therapy, with deferoxamine being the most common in both groups. Likewise, all patients were on blood transfusion therapy.

The serum ferritin level (ng/mL) for the intermediate and major groups was 2,266.71 ± 1,651.42 and 2,666.35 ± 1,703.33, respectively. The average tear film break-up time for the former group was 4.91 ± 1.44 seconds, while for the major group was 4.64 ± 1.73 seconds. For the control group, the average break-up time was 8.60 ± 1.45 seconds.

The measured average nerve length in the intermediate group was 3,277.28 ± 577 µm, while in the major group it was 3,112.71 ± 777.35 µm. This translated into a nerve density (mm/mm^2^) of 20.48 ± 3.61 and 19.45 ± 4.85 in the respective groups. Furthermore, nerve branch density (number of branches/mm^2^) was 36.66 ± 9.59 and 39.88 ± 14.44 in the intermediate and major cohorts, respectively. The nerve length, nerve density, and branch density for the control group were 4,319.87 ± 658.04 µm, 26.99 ± 4.11 mm/mm^2^, and 44.98 ± 3.16 number of branches/mm^2^, respectively. The Shapiro-Wilk test was used to assess the distribution of these densities, and both nerve length and density were found to be normally distributed.

One-way ANOVA was used to compare means between the three groups for nerve density and branch density, which had a statistically significant difference, [F (2, 101) = 34.517, p < 0.01] and [F (2, 101) = 5.984, p < 0.01], respectively. Moreover, the tear film break-up time was significantly different between the groups (Table 2).

**Table 2:** Corneal nerve parameters and tear break-up time in different groups. * Statistically significant.

Parameters	Intermediate group	Major group	Control group	P-value
	Mean ± standard deviation (range)			
Nerve density (mm/mm^2^)	20.48 ± 3.61 (14.15–25.14)	19.45 ± 4.85 (10.00–22.73)	26.99 ± 4.11 (19.56–35.21)	<0.001^*^
Branch density (branches/mm^2^)	36.66 ± 9.59 (14.58–58.33)	39.88 ± 14.44 (10.42–70.83)	44.98 ± 3.16 (37.60–51.23)	0.004^*^
Tear film break-up time (seconds)	4.89 ± 1.45 (3.00–9.00)	4.69 ± 1.74 (2.00–8.00)	8.77 ± 1.46 (6.00–11.00)	<0.001^*^

Post-hoc Bonferroni test showed higher mean values for the control group compared to the intermediate and major groups, and the results were statistically significant. Likewise, tear film break-up time was significantly higher among controls compared to both the thalassemic groups after the Bonferroni adjustment. The tear film break-up time was positively associated with corneal nerve density (Pearson’s r = 0.60, p < 0.001) and branch density (Pearson’s r = 0.28, p = 0.005). Serum ferritin levels did not correlate significantly with nerve characteristics (Table 3).

**Table 3:** Multiple group comparison (Bonferroni test) and correlation analysis. * Statistically significant.

Variable	Intermediate – major	Intermediate – control		Major – control
	Standardized mean difference (p-value)			
Nerve density	1.03 (1.00)	-6.51 (p < 0.001)^*^		-7.54 (p < 0.001)^*^
Branch density	-3.21 (0.620)	-8.31 (p = 0.003)^*^		-5.10 (p = 0.088)
Tear film break-up time	0.28 (1.00)	-3.68 (p < 0.001)^*^		-3.96 (p < 0.001)^*^
Pearson’s correlation				
Variable	Nerve density		Branch density	
Tear break-up time	r = 0.60, p < 0.001^*^		r = 0.28, p = 0.005^*^	
Serum ferritin levels	r = -0.07, p = 0.55		r = -0.09, p = 0.48	

## Discussion

This cross-sectional study utilized in vivo confocal microscopy (IVCM) to assess nerve characteristics in individuals with β-Th intermediate and β-Th major. Moreover, a comparative analysis was done with age-matched healthy controls. This study aimed to use CCM to quantify corneal nerve parameters as markers of peripheral neuropathy in thalassemia, which has been evaluated in the last few years with conflicting evidence using different methods. IVCM allows non-invasive examination of corneal nerves and has been effectively used in the early detection of neuropathies associated with diabetes and other systemic diseases. To our knowledge, peripheral neuropathy in thalassemia has not been studied using CCM.

In this study, it was observed that corneal nerve density (mm/mm^2^) was statistically higher in the control group (26.99 ± 4.11) compared to individuals with thalassemia major (19.45 ± 4.85) and thalassemia intermediate (20.48 ± 3.61). Likewise, branch density was higher in the control group, and the difference was statistically significant. However, the comparison of both these parameters did not yield statistically significant results when conducted between individuals with thalassemia major and intermediate.

The reduced density of nerves in patients with thalassemia compared to healthy controls suggests the presence of peripheral neuropathy. This is in contrast to some of the available evidence as both Bayhan et al. [10] and Işıkay et al. [24] did not observe features of peripheral neuropathy in thalassemia patients. However, there were differences in assessment between our study and the other two as the former used electrophysiological testing while the latter focused on large fibers for the assessment of neuropathy. Moreover, children were included in these studies, and there is evidence that neuropathy does not develop at least until adolescence. One of the more accurate methods for the assessment of peripheral neuropathy is through the assessment of small nerve fibers, and CCM allows for their non-invasive and in vivo assessment, which was used in this study.

There was no difference in nerve and branch densities between the thalassemia groups. Moreover, serum ferritin levels were not associated with any nerve parameters. This is in contrast to some other studies as it has been shown that neurological changes in thalassemia may be related to chronic hypoxia along with increased serum ferritin levels because of repeated transfusions. In our study, a comparative analysis of nerve characteristics with different modalities of iron chelation showed that there was no statistically significant difference in nerve and branch densities among patients receiving oral, intravenous, or a combination of both treatments. El-Tagui et al. [25] reported that iron overload is a risk factor for neuropathy. The lack of association between iron levels and chelation therapy with nerve densities in this study is in contrast to the work of El-Tagui et al. However, further work is required to evaluate its relation with small nerve fiber changes, keeping in view the limited sample of our study.

In our study, a positive correlation was observed between tear film break-up time and nerve and branch densities (r = 0.60, p < 0.001 and r = 0.28, p = 0.005, respectively). Dry eye disease has been associated with thalassemia [26], and this finding should not come as a surprise as corneal nerves have a central role in maintaining homeostasis of the ocular surface. Consequently, reduced corneal nerve density has a significant relationship with dry eye disease [27]. This is in line with our study, as has been discussed previously.

A study reported that neuropathy in thalassemia may be age-dependent and may manifest during adolescence [11]. This is in agreement with our work as respondents in this study had a mean age of 20.43 ± 8.73 and 17.65 ± 3.82 years in the intermediate and major groups, respectively, and reduced nerve density was observed in both these groups compared to age-matched healthy controls. It is suggested that up to 63% of thalassemia patients are expected to live beyond 50 years of age [28] in the current era of advanced medicine. This implies that the prevalence of neuropathic complications will increase in the coming years as treatment modalities improve, and it might be worthwhile to add peripheral neuropathic screening into basic examination protocols of β-Th patients. This requires the development of sensitive, reliable, and cost-effective methods to diagnose and monitor neuropathic changes, and CCM can be a reliable option, especially if it becomes widespread and mainstream in the near future. CCM has been reported as an extremely valuable tool in the assessment of neuropathies and multiple neurodegenerative diseases. Moreover, new morphologic parameters have also been developed to diagnose peripheral neuropathy, especially in association with diabetes [29].

There is a need to develop a diagnostic definition for peripheral neuropathy associated with β-Th because of the expected increase in the projected burden of this disease. Furthermore, its relationship with different iron chelation therapies and serum ferritin levels needs to be elucidated, with the potential role in estimating the requirement of chelation treatment while avoiding its toxicity. For this purpose, CCM studies with large sample sizes and standard quantification methods for corneal nerve assessments are warranted in the future. Our study points to a reduction in corneal nerve densities in thalassemia patients which may be a sign of peripheral neuropathy and warrants further studies using CCM.

This study had a few limitations pertaining to sample size and data collection. First, there was a very small sample size in the intermediate group. This is because these patients are very rarely diagnosed properly. Second, the findings in corneal nerves were not combined with electrophysiological testing and skin biopsy. Lastly, we used manual analysis through Image J instead of semi-automated software because of the lack of availability.

## Conclusions

The reduced corneal nerve density is an indication of peripheral neuropathy in patients with β-Th when examined through CCM. Further, studies with large sample sizes are warranted to validate the findings of this study. There were disparities in the results between our study and previous literature regarding the relationship between serum ferritin level and iron chelator use with neuropathic features. Nonetheless, it can be deduced that CCM can be a valuable tool as part of the comprehensive inventory of diagnostic and monitoring equipment in β-Th to assess neuropathic changes.
